# Prophylactic Regenerative Peripheral Nerve Interfaces for Prevention of Postamputation Pain: A Systematic Review and Meta-Analysis of Comparative Clinical Studies

**DOI:** 10.3390/jcm15155959

**Published:** 2026-07-30

**Authors:** Mark Gabriyanchik, Kirill Pirogov, Olesya Startseva, Kakhaber Meskhi, Igor Reshetov

**Affiliations:** 1 Department of Oncology, Radiotherapy and Reconstructive Surgery, Sechenov First Moscow State Medical University (Sechenov University), Moscow 119991, Russia; gabriyanchik_m_a@staff.sechenov.ru (M.G.); startseva_o_i@staff.sechenov.ru (O.S.); meskhi_k_t@staff.sechenov.ru (K.M.); reshetov_i_v@staff.sechenov.ru (I.R.); 2 Department for Business Development, Sechenov First Moscow State Medical University (Sechenov University), Moscow 119991, Russia

**Keywords:** regenerative peripheral nerve interface, RPNI, postamputation pain, phantom limb pain, symptomatic neuroma, active nerve management, opioid use, amputation, meta-analysis

## Abstract

**Background:** Chronic postamputation pain—phantom limb pain (PLP) and residual limb pain—is a major barrier to rehabilitation, with symptomatic neuroma formation a key peripheral contributor. The regenerative peripheral nerve interface (RPNI) provides a physiologic target for transected nerves. This review evaluated the effect of prophylactic RPNI, performed at the time of amputation, on pain-related outcomes. **Methods:** PubMed, Cochrane CENTRAL, ScienceDirect, SpringerLink, and MinhaBVS were searched through 23 November 2025 for comparative studies of prophylactic RPNI versus standard nerve management in major limb amputation. Primary outcomes were symptomatic neuroma incidence and PLP; chronic opioid use was a secondary outcome. Dichotomous outcomes were pooled using random-effects risk ratios (RR); for outcomes with zero events in one arm, the fixed-effect Peto odds ratio (OR) was used as the primary estimate. Risk of bias was assessed with ROBINS-I and certainty with GRADE. **Results:** Five comparative cohort studies (238 patients) were included; the primary pooled outcomes were derived from three of these cohorts (symptomatic neuroma, 196 patients; phantom limb pain, 181 patients), with two further studies contributing supportive descriptive data. Prophylactic RPNI markedly reduced symptomatic neuroma formation (0/97 vs. 20/99; Peto OR 0.11, 95% CI 0.05–0.29; *p* < 0.001; I^2^ = 0%) and PLP (RR 0.52, 95% CI 0.40–0.69; *p* < 0.001; I^2^ = 0%). Chronic opioid use at 12 months was also reduced (Peto OR 0.12, 95% CI 0.05–0.29; *p* < 0.001). Findings were consistent across pediatric, dysvascular, and oncologic populations. Certainty was low to very low. **Conclusions:** Prophylactic RPNI is associated with substantial, consistent reductions in symptomatic neuroma, phantom limb pain, and chronic opioid use after limb amputation. Despite reliance on low-certainty observational data, these consistent findings suggest that prophylactic RPNI may be a useful component of active nerve management; confirmation in prospective, adequately powered comparative studies is required.

## 1. Introduction

Limb amputation remains a major and growing source of long-term disability worldwide, driven by peripheral vascular disease, diabetes mellitus, and traumatic injury [1,2]. Despite advances in surgical technique and prosthetic technology, chronic postamputation pain continues to be a principal barrier to rehabilitation and quality of life. Phantom limb pain (PLP) and residual limb pain (RLP) are reported in up to 80% of adult amputees [3], and this burden extends to pediatric patients, in whom a substantial proportion develop chronic pain after limb loss. Management frequently requires prolonged opioid therapy, compounding the risk of dependence in trauma and oncologic populations. A key peripheral contributor to these syndromes is the symptomatic neuroma: in the absence of a physiologic distal target, axons regenerating from a transected nerve end undergo disorganized sprouting and form a terminal neuroma that generates neuropathic pain [4,5]. These pain phenotypes are mechanistically distinct. A symptomatic neuroma is a localized peripheral generator—a disorganized tangle of regenerating axons at the transected nerve stump. Residual (stump) limb pain is pain localized to the residual limb that may arise from a neuroma but also from soft-tissue, ischemic, or prosthetic-socket factors. Phantom limb pain, by contrast, is perceived in the missing limb and reflects both peripheral afferent input and maladaptive central (cortical) reorganization. Prophylactic RPNI acts at the peripheral node common to these mechanisms—the transected nerve end—which frames both its expected efficacy against neuroma formation and its more variable effect on the centrally mediated component of phantom limb pain.

Traditional intraoperative nerve management—traction neurectomy, ligation, or simple implantation of the nerve into surrounding tissue—is fundamentally passive and does not reliably prevent neuroma formation, yielding inconsistent pain control [4]. These shortcomings motivated the concept of “active nerve management,” which provides regenerating axons with a defined physiologic endpoint. Targeted muscle reinnervation (TMR) has become a widely adopted strategy and improves pain-related outcomes relative to standard neurectomy [6]; however, it requires expendable recipient motor nerves and microsurgical expertise, which may be unavailable in distal amputations, extensive trauma, or resource-limited settings. The regenerative peripheral nerve interface (RPNI) offers an alternative approach. In RPNI surgery, the transected nerve end is implanted into a free, devascularized autologous skeletal muscle graft, which subsequently revascularizes and is reinnervated, allowing organized axonal ingrowth and neuromuscular junction formation [5,7]. Unlike TMR, RPNI does not require identification of a recipient motor nerve and can be applied to virtually any peripheral nerve using muscle harvested from the operative field.

Although RPNI was originally developed to improve high-fidelity prosthetic control, the observation that it also prevents neuroma formation expanded its role into pain prevention [5,7]. Prophylactic RPNI performed at the time of amputation has since been associated with reduced symptomatic neuroma formation and lower rates of PLP across adult, dysvascular, pediatric, and oncologic populations [8,9,10,11]. To date, however, existing systematic reviews have been largely qualitative or have pooled RPNI together with TMR, obscuring the specific clinical effect of the RPNI construct, and none has incorporated the comparative evidence published in 2025 across diverse age groups and etiologies. We therefore conducted a systematic review and meta-analysis of comparative studies to quantify the effect of prophylactic RPNI, relative to standard passive nerve management, on the incidence of symptomatic neuroma, phantom limb pain, and chronic opioid use after major limb amputation.

## 2. Materials and Methods

This systematic review and meta-analysis was conducted and reported in accordance with the PRISMA 2020 statement [12] and the MOOSE guideline for meta-analyses of observational studies [13]. The review was not registered in PROSPERO; however, a written protocol specifying the search strategy, eligibility criteria, outcome domains, risk-of-bias tools, and analytical approach was prepared before screening and data extraction and is reproduced in Table S4. Two refinements made during the review are documented alongside the protocol in Table S4: the quantitative synthesis was restricted to comparative studies of prophylactic RPNI, and chronic opioid use was added as a secondary outcome once it was identified as a consistently reported endpoint. The absence of prospective registration is nonetheless acknowledged as a limitation.

### 2.1. Eligibility Criteria

We included comparative clinical studies (randomized or non-randomized cohort designs) of patients undergoing major upper- or lower-limb amputation, in which prophylactic RPNI performed at the time of amputation was compared with standard passive nerve management (e.g., traction neurectomy, ligation, or implantation of the nerve into surrounding tissue). To isolate the specific effect of the RPNI construct, studies combining RPNI with targeted muscle reinnervation (TMR) or other active nerve-management procedures—without separately reported RPNI data—were excluded. Single-arm therapeutic series without a concurrent comparator were not eligible for the quantitative synthesis. No language or publication-date restrictions were applied. Full inclusion and exclusion criteria are provided in Table S1.

### 2.2. Search Strategy and Data Extraction

A structured search of PubMed, the Cochrane Central Register of Controlled Trials (CENTRAL), ScienceDirect, SpringerLink, and the Virtual Health Library (MinhaBVS) was performed from inception to 23 November 2025. Search terms combined “regenerative peripheral nerve interface” or “RPNI” with concepts of amputation, neuroma, phantom limb pain, residual limb pain, and active nerve management. Reference lists of eligible articles were screened manually. Full search strings are provided in Table S2. Records were deduplicated and screened by title and abstract, followed by full-text assessment. Two reviewers independently performed study selection and data extraction; discrepancies were resolved by consensus; formal inter-rater agreement (e.g., Cohen’s κ) was not computed. Extracted variables included study design, demographics (age, sex, etiology), amputation level, follow-up duration, and outcome data. Where demographic data were reported as ranges, weighted means were calculated. Where event counts were not reported directly, they were derived from the reported group percentages and denominators and rounded to the nearest whole participant (Mohanty 2025 [10], control-arm neuroma events).

### 2.3. Outcomes

Primary outcomes were the incidence of symptomatic neuroma and phantom limb pain (PLP). The secondary outcome was chronic opioid use at 12 months. Additional comparative outcomes reported by single studies (severe residual limb pain, prosthesis use, pain-interference scores) were summarized descriptively.

### 2.4. Quality Assessment

Risk of bias was assessed for all included comparative studies using the ROBINS-I tool [14] across the domains of confounding, participant selection, classification of intervention, deviation from intended intervention, missing data, outcome measurement, and selection of the reported result. Certainty of evidence for each pooled outcome was rated using the GRADE framework [15] (Table S3).

### 2.5. Statistical Analysis

Dichotomous outcomes were pooled as risk ratios (RR) using the Mantel–Haenszel method under a random-effects (DerSimonian–Laird) model. For outcomes with zero events in one arm, the fixed-effect Peto odds ratio (OR) was used as the primary estimate, with the continuity-corrected Mantel–Haenszel RR reported as a sensitivity analysis, because the Peto method avoids the bias introduced by continuity corrections when events are rare.

Statistical heterogeneity was quantified with the I2 statistic. Given that fewer than ten studies contributed to each outcome, subgroup analyses, meta-regression, formal sensitivity (leave-one-out) analyses, funnel-plot assessment of publication bias, and trial sequential analysis were not performed, in accordance with Cochrane Handbook guidance; certainty was instead conveyed through GRADE. Analyses were conducted in Review Manager (RevMan), version 5.4 (The Cochrane Collaboration, Copenhagen, Denmark), and verified independently. A two-sided *p* < 0.05 was considered statistically significant.

## 3. Results

### 3.1. Study Selection and Baseline Characteristics

**Figure 1:**
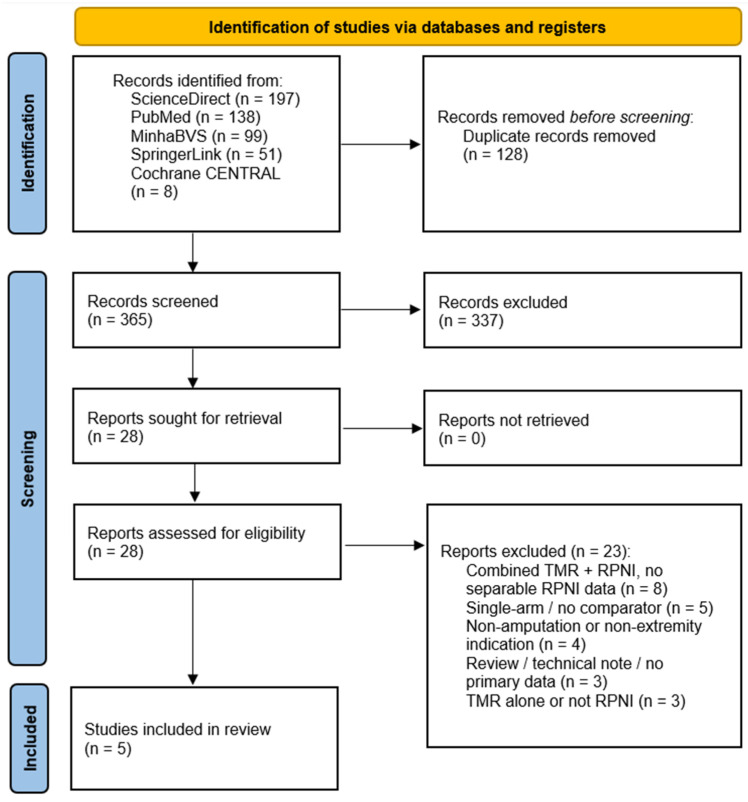
PRISMA flow diagram and study selection.

The database search identified 493 records; after removal of 128 duplicates, 365 records were screened and 28 reports were assessed in full text. Five comparative studies met the inclusion criteria and were included in the review (Figure 1) [8,9,10,11,16]. The pooled population comprised 238 patients (118 RPNI, 120 standard nerve management).

All five were non-randomized comparative cohorts (one matched-cohort design [8]) conducted between 2013 and 2025. The studies spanned a broad clinical spectrum, from pediatric amputees (mean age 14 years [10]) to elderly dysvascular patients with diabetes (mean age 68.6 years [9]), and included traumatic, dysvascular, and oncologic etiologies; two 2025 studies focused specifically on pediatric [10] and oncologic [11] populations. Amputations were predominantly of the lower limb (below- and above-knee), with upper-limb amputations represented in the oncologic cohort. Follow-up ranged from 6 to 26 months. Baseline characteristics are summarized in Table 1. Three studies [8,10,11] (Kubiak, Mohanty, Berberoglu) reported dichotomous outcomes eligible for pooling. The remaining two studies did not provide event data compatible with the pre-specified binary outcomes—Pejkova (2022) [9] reported severe residual-limb pain rather than symptomatic-neuroma incidence, and Lin (2023) [16] reported objective ultrasound neuroma dimensions rather than event counts—and were therefore summarized descriptively rather than pooled.

### 3.2. Risk of Bias

For non-randomized studies, the risk of bias was assessed using the Cochrane Collaboration’s tool for assessing the risk of bias in non-randomized studies of interventions (ROBINS-I). The ROBINS-I tool categorizes the risk of bias as low, moderate, serious, or critical. The matched-cohort study (Kubiak 2019) [8] was rated at moderate risk, partially mitigating confounding, whereas the smaller single-center cohorts (Pejkova 2022 [9], Lin 2023 [16]) were rated at serious risk. No major construct-related complications (e.g., muscle-graft necrosis) were reported in any study, and prophylactic RPNI was not associated with increased wound complications in dysvascular or diabetic patients.

### 3.3. Primary Outcomes

#### 3.3.1. Symptomatic Neuroma

**Figure 2:**
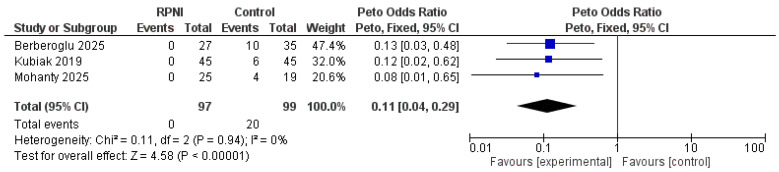
Forest plot of symptomatic neuroma incidence after prophylactic RPNI versus standard nerve management (Peto odds ratio, fixed-effect model). Blue squares represent the point estimate for each study, with square size proportional to study weight; horizontal lines indicate 95% confidence intervals; the black diamond represents the pooled estimate, with its width indicating the 95% confidence interval; the vertical line denotes the line of no effect. No neuroma events occurred in any RPNI arm (0/97 vs. 20/99); pooled Peto OR 0.11 (95% CI 0.05–0.29; *p* < 0.001), with no heterogeneity (I^2^ = 0%) [8,10,11].

Three comparative studies reported the incidence of symptomatic neuroma. No neuroma events occurred in any RPNI arm (0/97), compared with 20/99 in the standard-management arms. Prophylactic RPNI was associated with a significant reduction in symptomatic neuroma formation (Figure 2; Peto OR 0.11, 95% CI 0.05–0.29, *p* < 0.001), with no heterogeneity (I^2^ = 0%). This I^2^ value should be interpreted cautiously, as the statistic has low power to detect heterogeneity when only two or three small studies contribute to an estimate. A sensitivity analysis using the Mantel–Haenszel risk ratio with continuity correction was concordant (RR 0.07, 95% CI 0.01–0.38).

#### 3.3.2. Phantom Limb Pain

**Figure 3:**
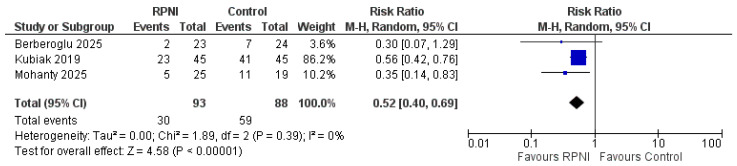
Forest plot of phantom limb pain incidence after prophylactic RPNI versus standard nerve management (Mantel–Haenszel risk ratio, random-effects model). Blue squares represent the point estimate for each study, with square size proportional to study weight; horizontal lines indicate 95% confidence intervals; the black diamond represents the pooled estimate, with its width indicating the 95% confidence interval; the vertical line denotes the line of no effect. Pooled RR 0.52 (95% CI 0.40–0.69; *p* < 0.001; I^2^ = 0%), with consistent effects across adult, pediatric, and oncologic cohorts [8,10,11].

The same three studies reported phantom limb pain. PLP occurred in 30/93 RPNI patients versus 59/88 controls (denominators for Berberoglu 2026 [11] differ across outcomes because each was reported for a different analyzable subset in the source: symptomatic neuroma for the full cohort [0/27 vs. 10/35], phantom limb pain for the subset with graded pain data [2/23 vs. 7/24], and 12-month opioid use for those with complete medication follow-up [0/17 vs. 15/28]). This corresponds to a significant reduction in PLP following prophylactic RPNI (Figure 3; RR 0.52, 95% CI 0.40–0.69, *p* < 0.001), with consistent effects across adult, pediatric, and oncologic populations (I^2^ = 0%).

### 3.4. Secondary Outcome: Chronic Opioid Use

**Figure 4:**
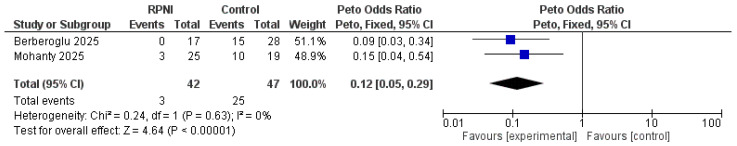
Forest plot of chronic opioid use at 12 months after prophylactic RPNI versus standard nerve management (Peto odds ratio, fixed-effect model). Blue squares represent the point estimate for each study, with square size proportional to study weight; horizontal lines indicate 95% confidence intervals; the black diamond represents the pooled estimate, with its width indicating the 95% confidence interval; the vertical line denotes the line of no effect. Pooled Peto OR 0.12 (95% CI 0.05–0.29; *p* < 0.001); Mantel–Haenszel risk ratio sensitivity analysis RR 0.18 (95% CI 0.06–0.53; I^2^ = 0%) [10,11].

Two studies reported chronic opioid use at 12 months. Long-term opioid dependence was lower in RPNI patients (3/42) than in controls (25/47); pooled analysis demonstrated a significant reduction (Figure 4; Peto OR 0.12, 95% CI 0.05–0.29, *p* < 0.001; sensitivity RR 0.18, 95% CI 0.06–0.53, I^2^ = 0%). In the oncologic cohort, all RPNI patients had discontinued opioids by 12 months, compared with 46% of controls [11].

### 3.5. Supportive Comparative Outcomes (Descriptive)

Outcomes reported by single studies were not pooled. In the dysvascular cohort [9], severe residual limb pain was absent after RPNI (0/14) versus 11/14 (78.6%) controls, successful prosthesis use was more frequent (12/14 vs. 3/14), and PROMIS pain-interference scores were lower (mean 52.6 vs. 68.5). In the only study using objective imaging [16], ultrasound-measured neuroma cross-sectional dimensions were significantly smaller after RPNI than after traditional amputation, providing objective corroboration of reduced neuroma formation.

### 3.6. Certainty of Evidence

GRADE certainty was low to very low across outcomes, reflecting the observational designs and small sample sizes, with downgrading for risk of bias and imprecision; the consistency of effect (I^2^ = 0%) and large magnitude for neuroma and opioid outcomes partially offset these limitations (Table S3).

## 4. Discussion

This systematic review and meta-analysis quantifies the effect of prophylactic RPNI, performed at the time of amputation, on the peripheral drivers of postamputation pain. Across five comparative cohorts (238 patients), prophylactic RPNI was associated with a near-complete absence of symptomatic neuroma formation (Peto OR 0.11), an approximately 50% reduction in phantom limb pain (RR 0.52), and a marked reduction in chronic opioid use (Peto OR 0.12). The consistency of effect across clinically diverse populations—pediatric, dysvascular, and oncologic—is encouraging, although the small number of studies and the low certainty of the evidence preclude firm conclusions about the generalizability of the effect.

The near-total absence of neuromas in RPNI arms is consistent with the construct’s biological rationale, which is supported by preclinical work showing that a free muscle graft implanted onto a transected nerve revascularizes and is reinnervated by organized axonal ingrowth without neuroma formation [17]. By providing regenerating axons with a physiologic distal target, RPNI is thought to halt the disorganized sprouting that otherwise produces a terminal neuroma. Because the diagnosis of symptomatic neuroma rests largely on clinical criteria and is therefore susceptible to assessment bias [18], the concordant finding from the only included study to use objective ultrasound imaging [16]—in which neuroma cross-sectional dimensions were significantly smaller after RPNI—provides important corroboration. The consistency of the effect in pediatric patients [10], who possess vigorous neuroregenerative capacity and are historically prone to neuroma recurrence, further suggests that the muscle-graft “sink” is sufficient to contain even aggressive axonal regeneration.

A central inference of this review concerns timing. Phantom limb pain is increasingly understood not as a purely peripheral phenomenon but as a consequence of maladaptive central plasticity, in which deafferentation drives a reorganization of the somatosensory cortex whose magnitude correlates with the severity of phantom symptoms [19,20]. Although RPNI was originally adopted to treat established neuroma pain, the magnitude and uniformity of the prophylactic effect support a “prevention-is-better-than-cure” paradigm: performing RPNI at the index amputation may attenuate the initial peripheral nociceptive barrage before it can entrench this cortical reorganization. Conversely, once long-standing pain and central sensitization are established, peripheral stabilization alone may be insufficient, as suggested by prospective therapeutic data in patients with pain of long duration [21]. This mechanistic asymmetry argues for incorporating RPNI into the primary amputation rather than reserving it for salvage of refractory pain.

The opioid-sparing signal is of particular clinical and public-health relevance. Approximately one in five opioid-naïve patients develops persistent opioid use after major limb amputation, with lower-limb amputation an independent risk factor [22]. Against this baseline, the association of prophylactic RPNI with a more than 80% lower risk of chronic opioid dependence (RR 0.18)—and complete opioid cessation by 12 months in the oncologic RPNI group—is clinically meaningful and supports prospective evaluation of RPNI as an opioid-sparing surgical strategy.

These findings have several implications for contemporary amputation practice. Because the benefit of RPNI is greatest when the construct is placed before a symptomatic neuroma and central sensitization become established, the strongest rationale is for routine prophylactic use at the index amputation rather than delayed salvage. The populations most likely to benefit are those at highest risk of disabling postamputation pain or prolonged opioid exposure—patients undergoing major limb amputation for trauma, dysvascular disease, or malignancy, and pediatric patients, in whom vigorous neuroregeneration predisposes to recurrent neuroma [10,23]. From a decision-making standpoint, RPNI adds little operative time and uses autologous muscle harvested from the operative field, so that, when a transected major nerve is encountered at amputation, prophylactic implantation can reasonably be considered a default in the absence of contraindications—recognizing that the supporting evidence remains of low certainty and that shared decision-making with the patient is appropriate.

RPNI is best understood as one component of a broader shift toward active nerve management and is complementary to—rather than in competition with—targeted muscle reinnervation (TMR). The two techniques share the principle of providing transected axons with a physiologic target but differ in their requirements and ideal indications. TMR coapts the amputated nerve to a nearby expendable motor nerve; it carries the strongest comparative evidence base, including randomized data for established neuroma pain [6] and prospective data when performed preemptively at amputation [24], and it additionally generates amplified myoelectric signals useful for advanced prosthetic control. However, TMR requires a suitable expendable recipient motor nerve, greater microsurgical expertise, and longer operative time, which may be impractical in distal amputations, extensive trauma, or resource-limited settings. RPNI instead implants the nerve end into a free autologous muscle graft and can be applied to virtually any peripheral nerve—including small sensory or digital nerves—using tissue from the operative field, with a short learning curve and minimal added morbidity [23]; its principal limitations are a smaller, lower-certainty evidence base and dependence on graft revascularization. Where a distal nerve stump is available, other options such as processed nerve allograft may further complement these techniques [25]. In practice, the choice is dictated by anatomy, the availability of recipient motor nerves, operative context, and surgeon experience, and the two procedures are increasingly used in combination.

Several evidence gaps must be addressed before prophylactic RPNI can be recommended for universal adoption. The current literature is dominated by retrospective, single-center cohorts with zero-event arms and short-to-moderate follow-up; adequately powered prospective multicenter studies and, where feasible, randomized comparative trials against TMR and standard neurectomy are needed to estimate effect sizes precisely and to isolate the independent contribution of RPNI. Outcome reporting should be standardized—using validated patient-reported instruments for phantom and residual limb pain, objective imaging of neuroma formation, and consistent case definitions of symptomatic neuroma—to permit reliable quantitative pooling. Longer follow-up is essential, because symptomatic neuromas and central sensitization may evolve over years, and formal cost-effectiveness analyses should determine whether the modest additional operative investment is offset by reductions in chronic pain, opioid use, and revision surgery. Until such data are available, the present findings are best regarded as strong but preliminary support for prophylactic RPNI applied within a shared decision-making framework.

## 5. Limitations

The evidence base remains predominantly retrospective and non-randomized (Level III), with an overall moderate-to-serious risk of bias related to residual confounding and subjective outcome assessment. The zero-event rate in RPNI arms, although clinically striking, required use of the Peto odds ratio and continuity-corrected sensitivity analyses; the resulting estimates should be interpreted as strong but imprecise. Several comparative outcomes derived from single studies (severe residual limb pain, prosthesis use, pain interference) were summarized descriptively and not pooled. The oncologic cohort used a historical control group, introducing potential era bias: controls were drawn from an earlier era within the study period (2010–2023), over which perioperative and pain-management practices may have evolved, and the precise interval between the control and RPNI periods was not reported in an extractable form. A substantial proportion of the included evidence originates from a single high-volume center, raising the possibility of partially overlapping populations. Finally, the review was not prospectively registered. These limitations temper the certainty of the pooled estimates, which were correspondingly rated low to very low under GRADE. Moreover, because each pooled outcome was informed by only two or three studies, the apparent absence of statistical heterogeneity (I2 = 0%) does not exclude clinically meaningful between-study variability. In addition, three of the five cohorts derive from a single center; although the enrollment windows of Kubiak (2019; 2013–2017) [8] and Mohanty (2025; 2000–2021) [10] overlap in calendar time, the two cohorts are drawn from non-overlapping age strata—adult amputees (mean age 46 years) and pediatric patients aged 8–21 years, respectively—so that a given patient could not have contributed to both. Individual-level data were not available to us and overlap therefore cannot be formally excluded, although substantial duplication is implausible on the basis of the published eligibility criteria. Symptomatic-neuroma case definitions were applied as reported by each study rather than harmonized, and follow-up ranged from 6 to 26 months; because symptomatic neuromas may declare late, shorter-duration data could underestimate their incidence, although all studies contributing to the pooled neuroma estimate had at least 12 months of follow-up. Finally, as an unregistered review, post-hoc influences on eligibility or outcome selection cannot be entirely excluded, notwithstanding that the protocol and outcomes were fixed before data extraction. The Peto odds ratio can be biased when treatment effects are large or group sizes are markedly unbalanced; however, the close concordance between the Peto OR, the Mantel–Haenszel risk ratio, and the random-effects estimate supports the robustness of the pooled effect [26].

## 6. Conclusions

Across diverse amputee populations, prophylactic RPNI performed at the time of amputation was associated with a substantial and consistent reduction in symptomatic neuroma formation, phantom limb pain, and chronic opioid use, with no detectable heterogeneity. Although the evidence is observational and of limited certainty, the magnitude and uniformity of effect, together with a favorable safety profile and broad technical applicability, suggest that prophylactic RPNI may be a valuable component of contemporary active nerve management, pending confirmation by higher-quality evidence. Adequately powered prospective and comparative studies are warranted to confirm these findings and to define the role of RPNI relative to other nerve-management techniques.

## Figures and Tables

**Table 1:** Baseline characteristics of the five included comparative studies.

Characteristics	Kubiak 2019 [8]	Pejkova 2022 [9]	Lin 2023 [16]	Mohanty 2025 [10]	Berberoglu 2026 [11]
Design	Retrospective matched cohort	Retrospective comparative	Retrospective comparative cohort	Retrospective comparative cohort	Retrospective comparative cohort
Country, period	USA (Michigan), 2013–2017	North Macedonia, 2019–2020	China, 2018–2021	USA (Michigan), 2000–2021	USA, 2010–2023
No. RPNI/Control	45/45	14/14	7/7	25/19	27/35 *
Male, *n* (%)	65 (72%)	19 (68%)	11 (79%)	33 (75%)	38 (61%)
Age, y (RPNI/Control)	46 ± 17.4/ 44.2 ± 13.8	68.6 (49–85)	25.9 ± 18.7/ 20.1 ± 13.9	14 ± 5/ 14 ± 6	49.8 ± 19.0/ ~50.5 ± 19.0
Population	Adult major-limb amputees	Diabetic lower-limb amputees	Oncologic lower-limb amputees	Pediatric (8–21 y)	Oncologic (upper & lower limb)
Follow-up, months	11.9	11.3	6	20.5	26.3
Contribution	Pooled (neuroma, PLP)	Descriptive (severe RLP, prosthesis, PROMIS)	Descriptive (ultrasound neuroma size)	Pooled (neuroma, PLP, opioids)	Pooled (neuroma, PLP, opioids)

Values are mean ± SD or mean (range) unless stated. * Denominators for Berberoglu 2026 [11] differ by outcome (symptomatic neuroma 27/35, full cohort; phantom limb pain 23/24; 12-month opioid use 17/28), reflecting outcome-specific analyzable subsets in the source. Amputation levels: Kubiak, BKA ~83%/AKA ~21%; Pejkova, BKA and AKA; Lin, sciatic/tibial/common peroneal; Mohanty, predominantly BKA; Berberoglu, AKA/BKA and hemipelvectomy (33% upper limb). Sex is given as the cohort total. RPNI, regenerative peripheral nerve interface; BKA, below-knee amputation; AKA, above-knee amputation; RLP, residual limb pain; PLP, phantom limb pain; PROMIS, Patient-Reported Outcomes Measurement Information System.

## Data Availability

The original contributions presented in this study are included in the article/Supplementary Materials. Further inquiries can be directed to the corresponding author.

## Supplementary Materials

The following supporting information can be downloaded at: https://www.mdpi.com/article/10.3390/jcm15155959/s1, PRISMA 2020 checklist [12]; Table S1: Inclusion and exclusion criteria; Table S2: Adjusted search strategies across electronic databases (as of 23.11.2025); Table S3: GRADE evidence profile: prophylactic RPNI for postamputation pain outcomes; Figure S1. ROBINS-I risk-of-bias assessment of the included comparative studies; Table S4: A priori review protocol and protocol refinements.

## Abbreviations

The following abbreviations are used in this manuscript:

RPNI	Regenerative peripheral nerve interface
TMR	Targeted muscle reinnervation
PLP	Phantom limb pain
RLP	Residual limb pain
PROMIS	Patient-Reported Outcomes Measurement Information System
RR	Risk ratio
OR	Odds ratio
CI	Confidence interval
ROBINS-I	Risk Of Bias In Non-randomised Studies of Interventions
GRADE	Grading of Recommendations Assessment, Development and Evaluation
PRISMA	Preferred Reporting Items for Systematic Reviews and Meta-Analyses
CENTRAL	Cochrane Central Register of Controlled Trials
