# Supplementary material for: Prophylactic Regenerative Peripheral Nerve Interfaces for Prevention of Postamputation Pain: A Systematic Review and Meta-Analysis of Comparative Clinical Studies
Source: J Clin Med. 2026 Jul 30;15(15):5959. doi: 10.3390/jcm15155959 (PMC13466367; doi:10.3390/jcm15155959)
Supplement: Supplementary file 1 [file jcm-15-05959-s001.zip › Table S1.pdf]

Table S1. Inclusion and exclusion criteria.

Research question: "What is the effect of prophylactic regenerative peripheral nerve interfaces (RPNI), performed at the time of amputation, on postamputation pain outcomes compared with standard nerve management?"

Criterion	Inclusion	Exclusion
**Participants**	• Adults and pediatric patients undergoing major upper- or lower-limb amputation	• Animal studies<br>• Cadaveric or purely biomechanical studies<br>• Studies not involving limb amputation
**Intervention**	• Prophylactic RPNI performed at the time of index amputation<br>• Studies clearly describing the RPNI construct	• Therapeutic RPNI for established pain without a concurrent comparator<br>• Combined procedures where RPNI effects could not be isolated (e.g., RPNI + TMR without separate analysis)<br>• TMR alone without RPNI
**Comparators**	• Standard passive nerve management (traction neurectomy, ligation, nerve burying/relocation)<br>• Concurrent or historical control groups	• Studies without a concurrent comparator group (single-arm series)
**Outcomes**	Primary: incidence of symptomatic neuroma; incidence of phantom limb pain (PLP)<br>Secondary: chronic opioid use; (descriptive) residual limb pain severity, prosthesis use, pain-interference scores	• Studies not reporting pain-related outcomes
**Study Design**	• Randomized controlled trials (if available)<br>• Prospective or retrospective comparative cohort studies<br>• Case–control studies<br>Search date: 23 November 2025; published articles and preprints; no language restriction	• Single-arm series and pre–post studies without a concurrent comparator<br>• Editorials, commentaries, letters<br>• Narrative reviews; systematic reviews/meta-analyses<br>• Abstract-only publications; case reports
