# Supplementary material for: Prophylactic Regenerative Peripheral Nerve Interfaces for Prevention of Postamputation Pain: A Systematic Review and Meta-Analysis of Comparative Clinical Studies
Source: J Clin Med. 2026 Jul 30;15(15):5959. doi: 10.3390/jcm15155959 (PMC13466367; doi:10.3390/jcm15155959)
Supplement: Supplementary file 1 [file jcm-15-05959-s001.zip › Table S2.pdf]

Table S2. Adjusted search strategies across electronic databases (as of 23.11.2025)

**Database**	**Search Query (simplified for reporting)**	**Records Retrieved (n)**
**PubMed**	("Regenerative Peripheral Nerve Interface" OR RPNI) AND (pain OR neuroma OR "phantom limb" OR "residual limb" OR prosthe* OR "targeted muscle reinnervation" OR TMR)	**138**
**ScienceDirect**	("regenerative peripheral nerve interface" OR RPNI) AND (pain OR neuroma OR "phantom limb" OR "residual limb" OR prosthe* OR "targeted muscle reinnervation" OR TMR)	**197**
**Cochrane Library (CENTRAL)**	("regenerative peripheral nerve interface" OR RPNI) AND (pain OR neuroma OR "phantom limb" OR "residual limb")	**8**
**SpringerLink**	("regenerative peripheral nerve interface" OR RPNI) AND (pain OR neuroma OR "phantom limb" OR "residual limb")	**51**
**MinhaBVS (Virtual Health Library)**	("regenerative peripheral nerve interface" OR RPNI) AND (pain OR neuroma OR "phantom limb")	**99**
