# Supplementary material for: Prophylactic Regenerative Peripheral Nerve Interfaces for Prevention of Postamputation Pain: A Systematic Review and Meta-Analysis of Comparative Clinical Studies
Source: J Clin Med. 2026 Jul 30;15(15):5959. doi: 10.3390/jcm15155959 (PMC13466367; doi:10.3390/jcm15155959)
Supplement: Supplementary file 1 [file jcm-15-05959-s001.zip › Table_S3_GRADE.pdf]

Table S3. GRADE evidence profile: prophylactic RPNI for postamputation pain outcomes.

Outcome	No. of studies (Design)	Risk of bias	Inconsistency	Indirectness	Imprecision	Publication bias	No. of patients (RPNI/Ctrl)	Effect estimate (95% CI)	Certainty
**Prophylactic RPNI vs. standard nerve management**									
Symptomatic neuroma	3 (Obs.)	Serious[1]	No serious (I^2 = 0%)	No serious	No serious	Not assessed[2]	0/97 vs 20/99	Peto OR 0.11 (0.05–0.29)	⊕⊕⊖⊖ Low[3]
Phantom limb pain	3 (Obs.)	Serious[1]	No serious (I^2 = 0%)	No serious	No serious	Not assessed[2]	30/93 vs 59/88	RR 0.52 (0.40–0.69)	⊕⊕⊖⊖ Low[3]
Chronic opioid use (12 mo)	2 (Obs.)	Serious[1]	No serious (I^2 = 0%)	No serious	Serious[4]	Not assessed[2]	3/42 vs 25/47	Peto OR 0.12 (0.05–0.29)	⊕⊖⊖⊖ Very Low

Quality ratings: High (⊕⊕⊕⊕), Moderate (⊕⊕⊕⊖), Low (⊕⊕⊖⊖), Very Low (⊕⊖⊖⊖).

[1] All included studies were non-randomized observational designs; starting certainty for observational evidence is Low. Risk of bias was moderate to serious (ROBINS-I), mainly from residual confounding and non-blinded outcome assessment.

[2] Publication bias was not formally assessed (funnel plots not performed) because fewer than 10 studies contributed to each outcome.

[3] Upgraded one level for large magnitude of effect (Peto OR < 0.2 / RR < 0.5), per GRADE guidance for observational studies with large effects.

[4] Downgraded for imprecision: only two studies contributed and the confidence interval, although significant, is wide.

Abbreviations: CI, confidence interval; GRADE, Grading of Recommendations Assessment, Development and Evaluation; I^2, heterogeneity statistic; Obs., observational; OR, odds ratio; RPNI, regenerative peripheral nerve interface; RR, risk ratio.
