# Supplementary material for: Prophylactic Regenerative Peripheral Nerve Interfaces for Prevention of Postamputation Pain: A Systematic Review and Meta-Analysis of Comparative Clinical Studies
Source: J Clin Med. 2026 Jul 30;15(15):5959. doi: 10.3390/jcm15155959 (PMC13466367; doi:10.3390/jcm15155959)
Supplement: Supplementary file 1 [file jcm-15-05959-s001.zip › Table_S4_protocol.pdf]

Table S4. A priori review protocol and protocol refinements.

The protocol was prepared before screening and data extraction. Refinements made during the conduct of the review are documented in the right-hand column in accordance with PRISMA 2020 item 24c.

Protocol element	Pre-specified in protocol	As implemented (refinement and rationale)
**Search strategy**	(“regenerative peripheral nerve interface” OR RPNI) AND (pain OR neuroma OR “phantom limb” OR “residual limb” OR prosthe* OR “targeted muscle reinnervation” OR TMR)	Applied as specified across PubMed, Cochrane CENTRAL, ScienceDirect, SpringerLink and MinhaBVS (full strings in Table S2).
**Population**	Patients with amputation and/or neuroma-related pain; upper and lower limb; level of amputation recorded.	Applied as specified. Pediatric cohorts were retained when comparative pediatric data were identified (Mohanty 2025); age was not applied as an exclusion criterion.
**Intervention**	RPNI, therapeutic or prophylactic; number and type of constructs recorded.	Refinement: quantitative synthesis restricted to prophylactic RPNI performed at the index amputation, because the therapeutic evidence consisted solely of single-arm pre–post series without a concurrent comparator.
**Comparators**	Standard care (neurectomy / burying / relocation) and/or TMR; studies with clear comparator groups preferred.	Refinement: a concurrent comparator was made a requirement for inclusion in the synthesis; single-arm series were not pooled.
**Primary outcomes**	PLP, RLP, neuroma pain (event rates) and pain intensity (VAS/NRS).	Symptomatic neuroma and phantom limb pain retained as primary. Residual limb pain and pain intensity were reported descriptively rather than pooled, owing to incompatible reporting formats across studies.
**Secondary outcomes**	Prosthesis wear/use; complications.	Prosthesis use and complications reported descriptively. Refinement: chronic opioid use at 12 months was added as a secondary outcome once identified as a consistently reported endpoint in the comparative cohorts.
**Eligible designs**	RCT, cohort, case–control, pre–post cohorts ($n \geq 5$); case series permitted for sensitivity analysis.	Refinement: restricted to comparative designs. No randomized trials were identified; pre–post and case-series designs were not synthesized quantitatively.
**Risk-of-bias tools**	RoB 2 (randomized); ROBINS-I (non-randomized); NIH tool (case series, sensitivity only).	ROBINS-I applied to all five included comparative cohorts (Figure S1). RoB 2 and the NIH tool were not required, as no randomized trials or case series were retained.
**Effect measures**	Binary: RR/OR. Continuous: MD or SMD (Hedges g). Pre–post: change scores; if unavailable, pre/post with assumed $r = 0.5$ (sensitivity $r = 0.3–0.7$).	Binary outcomes pooled as Mantel–Haenszel RR under a random-effects model; the fixed-effect Peto OR used where one arm had zero events. Pre–post methods were not required following exclusion of single-arm data.
**Missing standard deviations**	Impute from CI/SE/p-values or IQR; $SD = SE \times \sqrt{n}$; $SD = (\text{upper} - \text{lower})/(2 \times 1.96)$ for a 95% CI.	Applied as specified where required. Where event counts were not reported directly, they were derived from reported percentages and denominators.
**Heterogeneity**	Random-effects model; I^2, τ^2. Predefined subgroups: level of amputation, prophylactic vs therapeutic, comparator type, follow-up.	Random-effects (DerSimonian–Laird) with I^2 applied as specified. Subgroup analyses were not performed because fewer than ten studies contributed to each outcome (Cochrane Handbook guidance).
**Publication bias**	Funnel plot and Egger test if $k \geq 10$; rationale documented if not applicable.	Applied as specified: not performed, as fewer than ten studies contributed to each outcome; rationale stated in §2.5.

CI, confidence interval; MD, mean difference; NRS, numerical rating scale; PLP, phantom limb pain; RLP, residual limb pain; RoB 2, Cochrane risk-of-bias tool for randomized trials; ROBINS-I, Risk Of Bias In Non-randomised Studies of Interventions; RPNI, regenerative peripheral nerve interface; RR, risk ratio; SD, standard deviation; SE, standard error; SMD, standardized mean difference; TMR, targeted muscle reinnervation; VAS, visual analogue scale.
